# Supplementary material for: JWST Observations of Segregated 12CO2 and 13CO2 Ices in Protostellar Envelopes
Source: ACS Earth Space Chem. 2025 Jul 17;9(8):1992–2003. doi: 10.1021/acsearthspacechem.5c00037 (PMC12376187; doi:10.1021/acsearthspacechem.5c00037)
Supplement: Supplementary file 1 [file sp5c00037_si_001.pdf]

Supporting Information Available

Supporting information: Additional figures including continuum fittings in the 15 μm region, alternative band profile fittings of the CO_2 ice bands with different laboratory spectra, band profile fitting of the 3.5 μm methanol feature and tables containing properties of the two sources and the FWHM and central positions of the Gaussian curves fitted to the $^{13}CO_2$ ice band.

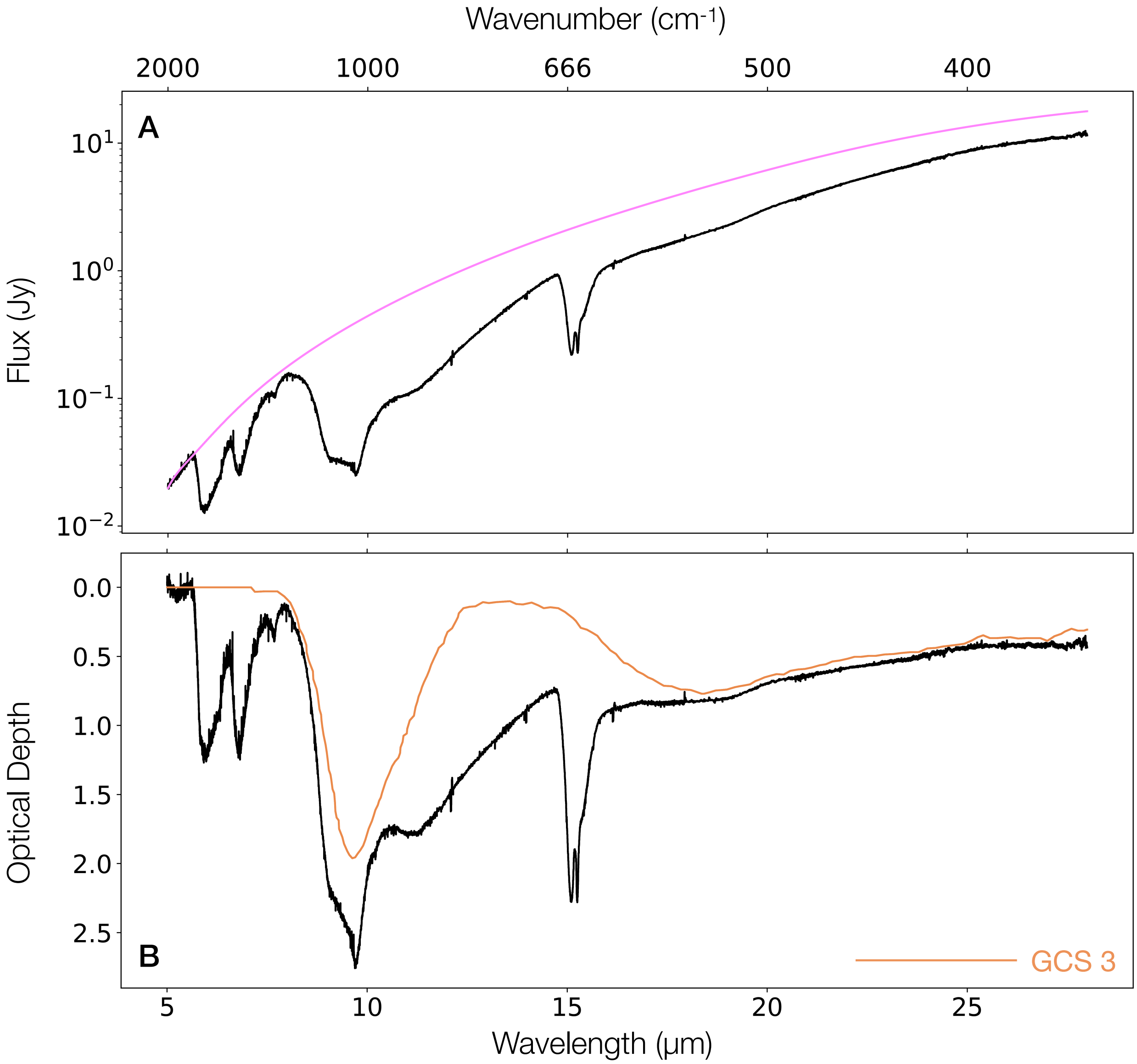

Figure 7: Continuum determination for Per-emb 35. Panel A shows the global continuum fitted over the 5 - 28 μm region (pink). Panel B shows the spectrum of GCS 3 fitted over the continuum subtracted spectrum (orange).

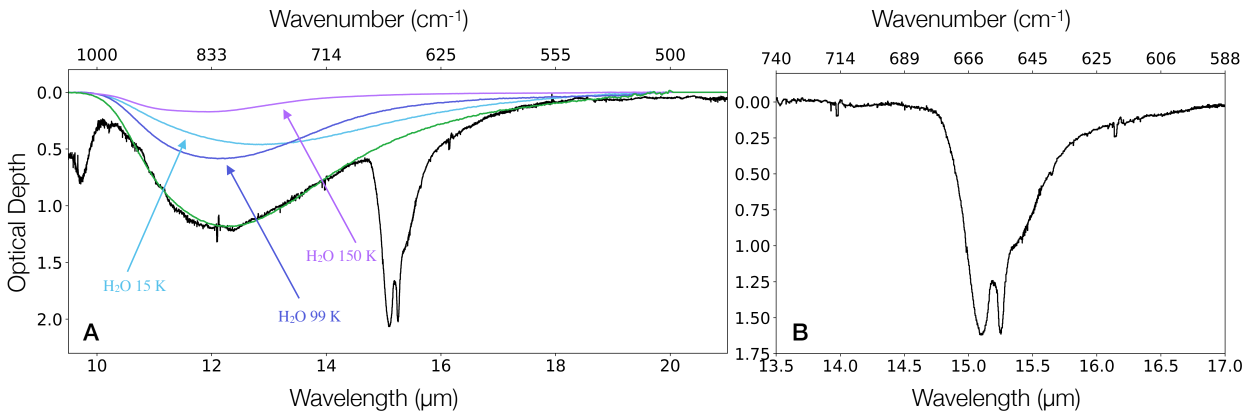

Figure 8: Subtraction of the water libration mode in Per-emb 35. Panel A shows the spectrum of water ice at different temperature fitted over the silicate subtracted spectrum. Panel B shows the final spectrum on optical depth scale.

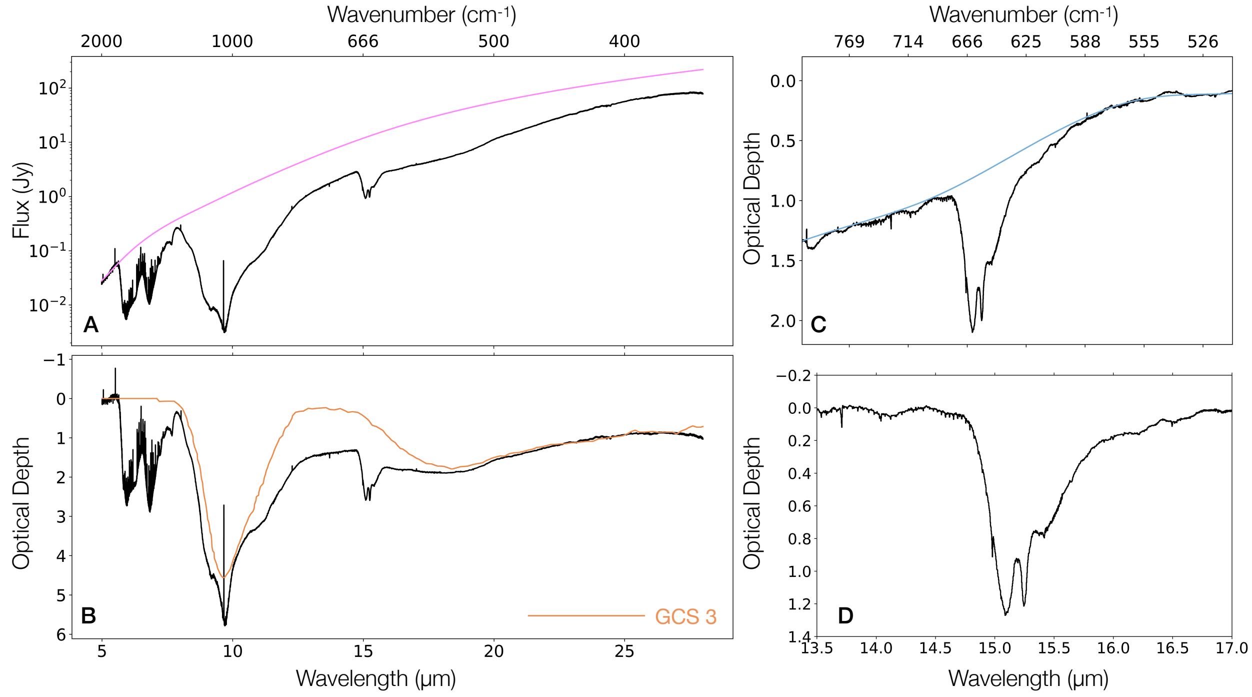

Figure 9: Continuum determination for IRAS 20126. Panel A shows the global continuum fitted over the 5 - 28 μm region (pink). Panel B shows the spectrum of GCS 3 fitted over the continuum subtracted spectrum (orange). Panel C shows the local continuum fitted over the silicate subtracted spectrum to simulate the wing of the water libration mode. Panel D shows the final spectrum on optical depth scale.

Table 6: Source properties.

Source	RA	Dec	L ($L_\odot$)	Distance (pc)
IRAS 20126	20:14:26.04	41:13:32.43	10^4	1550
Per 35	3:28:37.093	31:13:30.83	9.3	293

Table 7: Properties of the spectral features comprising the $^{13}CO_2$ band of IRAS 20126.

Component	Peak Position (cm^{-1})	FWHM (cm^{-1})
Long-wavelength	2275.72 $\pm$ 0.1	6.6 $\pm$ 0.1
Middle	2278.90 $\pm$ 0.4	9.0 $\pm$ 0.6
Short-wavelength	2282.91 $\pm$ 0.01	3.5 $\pm$ 0.1

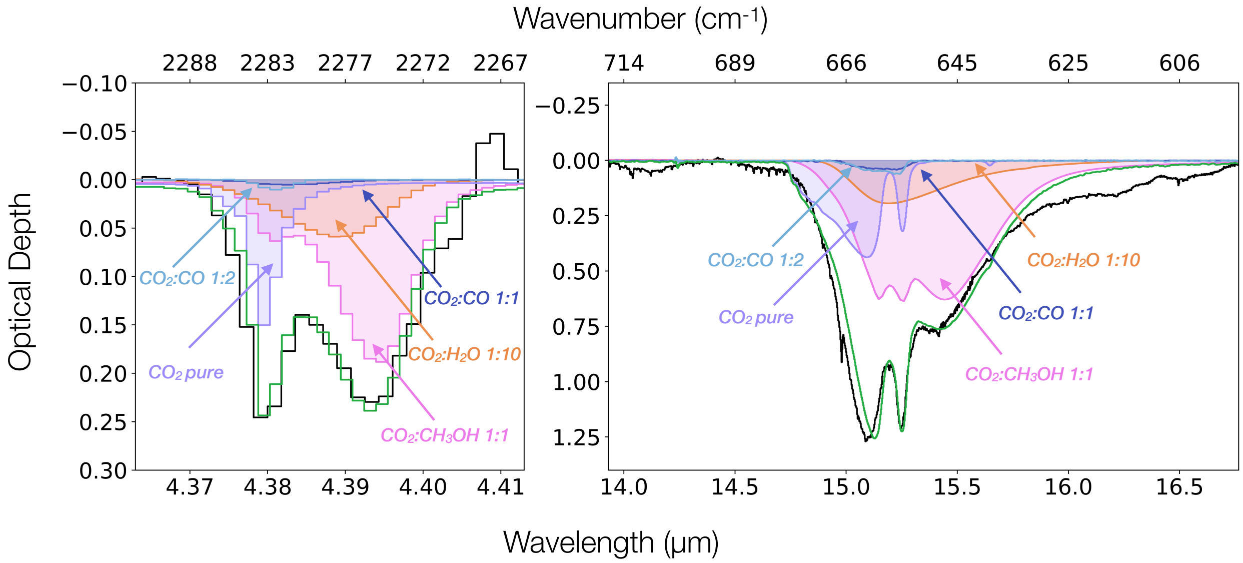

Figure 10: Alternative band profile analysis of IRAS 20126. Left: Decomposition of the 4.39 μm $^{13}CO_2$ band. Right: Decomposition of the 15.2 μm $^{12}CO_2$ band. The purple, pink, orange, light blue and dark blue shaded areas correspond to the pure CO_2 80 K, CO_2:CH_3OH 1:1 115 K, CO_2:H_2O 1:10 10 K, CO_2:CO 1:2 25 K and CO_2:CO 1:1 15 K component, respectively. Finally the green line shows the sum of all the components. The poor fit at 16.2 μm and 16.5 μm is likely due to absorption features of crystalline silicates.

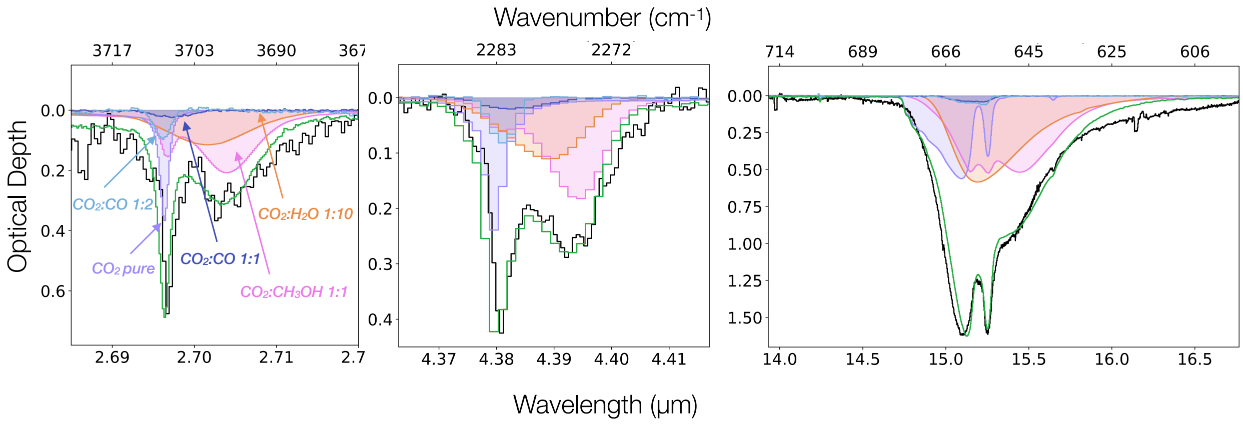

Figure 11: Alternative band profile analysis of Per-emb 35. Left: Decomposition of the 4.39 μm $^{13}CO_2$ band. Right: Decomposition of the 15.2 μm $^{12}CO_2$ band. The purple, pink, orange, light blue and dark blue shaded areas correspond to the pure CO_2 80 K, CO_2:CH_3OH 1:1 115 K, CO_2:H_2O 1:10 10 K, CO_2:CO 1:2 25 K and CO_2:CO 1:1 15 K component, respectively. Finally the green line shows the sum of all the components. The poor fit at 16.2 μm and 16.5 μm is likely due to absorption features of crystalline silicates.

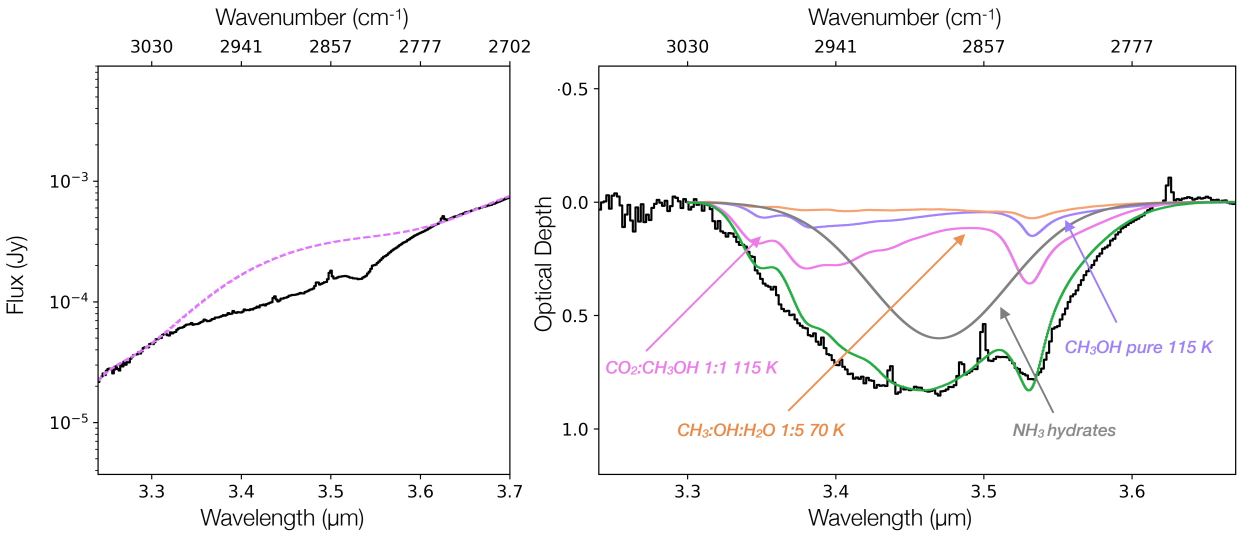

Figure 12: Continuum determination (left) and spectral decomposition (right) of the 3.5 CH_3OH feature. The purple, pink, orange, and gray lines correspond to spectra of pure CH_3OH 115 K, CO_2:CH_3OH 1:1 115 K, H_2O:CH_3OH 5:1 70 K and a Gaussian representing the ammonia hydrates in this region, respectively. The H_2O:CH_3OH and pure CH_3OH spectra were obtained from Slavicinska et al.[33] and the properties of the Gaussian curve are based on the results presented in Boogert et al.[53].
